# Nuclear Receptor Interaction Protein (NRIP) expression assay using human tissue microarray and immunohistochemistry technology confirming nuclear localization

**DOI:** 10.1186/1756-9966-27-25

**Published:** 2008-08-02

**Authors:** Chih-Ping Han, Ming-Yung Lee, Shu-Ling Tzeng, Chung-Chin Yao, Po-Hui Wang, Ya-Wen Cheng, Show-Li Chen, Teresa S Wu, Yeu-Sheng Tyan, Lai-Fong Kok

**Affiliations:** 1 Department of Obstetrics and Gynecology, Chung-Shan Medical University Hospital, Taichung, Taiwan; 2 Institute of Medicine, Chung-Shan Medical University, Taichung, Taiwan; 3 Clinical Trial Center, Chung-Shan Medical University Hospital, Taichung, Taiwan; 4 Department of Surgery, Chung-Shan Medical University Hospital, Taichung, Taiwan; 5 Graduate Institute of Microbiology, College of Medicine, National Taiwan University, Taipei, Taiwan; 6 Department of Emergency Medicine, Graduate Medical Education, Orlando Regional Medical Center, Orlando, Florida, USA; 7 Department of Medical Imaging, Chung-Shan Medical University Hospital, Taichung, Taiwan; 8 Department of Medical Imaging and Radiological Science, Chung-Shan Medical University, Taichung, Taiwan; 9 Department of Pathology, China Medical University Hospital, Taichung, Taiwan

## Abstract

**Background:**

A novel human nuclear receptor interaction protein (NRIP) has recently been discovered by Chen SL et al, which may play a role in enhancing the transcriptional activity of steroid nuclear receptors in prostate (LNCaP) and cervical (C33A) cancer cell lines. However, knowledge about the biological functions and clinical implications of NRIP, is still incomplete. Our aim was to determine the distribution of NRIP expression and to delineate the cell types that express NRIP in various malignant tumors and healthy non-pathological tissues. This information will significantly affect the exploration of its physiological roles in healthy and tumor cells.

**Methods:**

By using tissue microarray (TMA) technology and an anti-NRIP monoclonal antibody immunohistochemical (IHC) survey, NRIP expression was examined in 48 types of tumors and in a control group of 48 matched or unmatched healthy non-neoplastic tissues.

**Results:**

Our survey results showed that ten cases were revealed to express the NRIP in six malignancies (esophageal, colon, breast, ovarian, skin, and pancreatic cancers), but not all of these specific tumor types consistently showed positive NRIP expression. Moreover, malignant tumors of the stomach, prostate, liver, lung, kidney, uterine cervix, urinary bladder, lymph node, testis, and tongue revealed no NRIP expression. Among the control group of 48 matched and unmatched non-neoplastic tissues, all of them demonstrated IHC scores less than the cut-off threshold of 3. In addition, ten cores out of thirty-six carcinomatous tissues revealed positive NRIP expression, which indicated that NRIP expression increases significantly in carcinoma tissue cores, comparing to the matched controlled healthy tissues.

**Conclusion:**

This is the first study to use a human TMA and IHC to validate the nuclear localization for this newly identified NRIP expression. In considering the use of NRIP as a potential diagnostic tool for human malignancies survey, it is important to note that NRIP expression carries a sensitivity of only 23%, but has a specificity of 100%. There is also a significant difference in positive NRIP expression between primary carcinomatous tissues and matched controlled healthy tissues. Although further large-scale studies will merit to be conducted to evaluate its role as a potential adjunct for cancer diagnosis, data from this study provides valuable references for the future investigation of the biological functions of NRIP in humans.

## Background

Chen et al. cloned a novel human nuclear receptor interaction protein (NRIP) gene (GenBankTM accession numbers AY766164 [GenBank] and AAX09330 [GenBank]) and deposited it in the National Center for Biotechnology Information (NCBI) with an unknown function in 2005. They characterized this human novel gene (NRIP) by assaying its sub-cellular location in cultured 293T cells, examining its interaction with some nuclear receptors (such as AR and GR), and evaluating its trans-activation activity in distinct promoters. Their results indicated that: (1) NRIP contains 860 amino acids and its expression is in the cell nucleus; (2) NRIP binds to both AR and GR and functions as a nuclear receptor co-activator, so it may be a transcriptional cofactor of steroid receptors; and (3) by using a specific NRIP siRNA targeting sequence, it could knock down endogenous and exogenous NRIP gene expression, resulting in significantly diminished cell proliferation in prostate (LNCaP) and cervical (C33A) cancer cells [1,2]. Although NRIP may function to enhance the transcriptional activity of nuclear receptors, the precise physiologic role of NRIP is still unclear.

Tissue microarray (TMA) technology has enabled researchers to investigate multiple specimens simultaneously with immunohistochemical (IHC) technology. This results resulting in a dramatic reduction of time and cost compared with conventional histopathologic research techniques. TMA has become a popular tool for tissue-based research, because it allows for massive acceleration of studies correlating molecular in situ findings with clinico-pathological information. This approach has become specifically useful in surveys of tumor populations where it can be utilized to analyze the functions of newly identified genes in both healthy and neoplastic human tissues in both a comprehensive and efficient manner [3-5]. In order to explore the relationship between NRIP expression and its biological functions, Chen et al developed monoclonal antibodies against NRIP [2]. We believe that further characterization of the subcellular localization of NRIP expression in various human tissues will significantly clarify its physiologic and pathologic roles throughout the body and help delineate its potential biological functions. This study is the first comprehensive survey of NRIP in combined multiple human malignancies, containing somatic, germ line, embryonic tumors and non-pathological controls in tissue microarray (TMA).

## Methods

**Figure 1:**
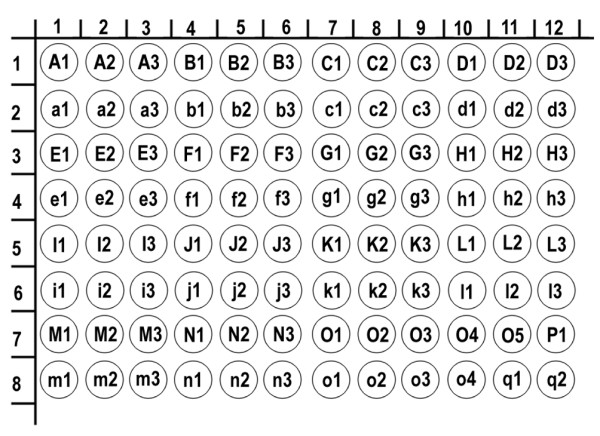
**US Biomax Inc Catalog No. BCN962 Microarray Panel Display (total 96 cores)**. A1-3/a1-3: Esophagus, B1-3/b1-3: Stomach, C1-3/c1-3: Colon, D1-3/d1-3: Prostate, E1-3/e1-3: Liver, F1-3/f1-3: Lung, G1-3/g1-3: Kidney, H1-3/h1-3: Breast, I1-3/i1-3: Uterine cervix, J1-3/j1-3: Ovary, K1-3/k1-3: Bladder, L1-3/l1-3: Lymph node, M1-3/m1-3: Skin, N1-3/n1-3: Pancreas, O1-5/o1-4: Testis, P1: Tongue, q1-2: Placenta.

We evaluated 48 tumor cores and 48 matched and unmatched non-neoplastic tissue samples using human tissue microarray technology (US Biomax Inc Catalog No. BCN962). (Figure 1) Tissue samples were arranged in 12 columns of 8 rows for a total of 96 individual cores (1 mm, 5 μm). All samples of this commercially derived tissue microarray (TMA), originated from different donors. Researches were blinded to the names and identities of the specimens and donors. Two board-certified pathologists (CP Han & LF Kao) re-confirmed the histopathologic features of all of the samples. Of the 48 tumor cores, only 44 contained tumor cells on histopathologic review. The other 4 clean samples were thought to be missing tumor components secondary to either inappropriate acquisition or a processing error during TMA construction.

The 48 cores of labeled neoplastic tissues included specimens from the esophagus, stomach, colon, prostate, liver, lung, kidney, breast, uterine cervix, ovary, urinary bladder, lymph node, skin, pancreas, testis and tongue. (Table 1) The control group of 48 cores of unmatched or matched non-neoplastic tissue included specimens from the esophagus, stomach, colon, prostate, liver, lung, kidney, breast, uterine cervix, ovary, urinary bladder, stomach lymph node, skin, pancrease, testis, and placenta. Immunohistochemistry and antigen retrieval methods were utilized in the same manner as described in previous literature [6]. Briefly, the tissue microarray slide, containing 96 cores of specimens (1 mm, 5 μm in each), was commercially derived from US Biomax Inc. All samples were washed in xylene to remove the paraffin and then rehydrated through serial dilutions of alcohol, followed by washings with a solution of PBS (pH 7.2). All subsequent washes were buffered via the same protocol. Treated sections were then placed in a citrate buffer (pH 6.0) and heated in a microwave for two 5-minute sessions. The samples were then incubated with a monoclonal anti-human NRIP antibody (kindly provided by SL Chen) for 60 minutes at 25°C. The conventional streptavidin peroxidase method (DAKO, LSAB Kit K675, Copenhagen, Denmark) was performed for signal development and the cells were counter-stained with hematoxylin. Negative controls were obtained by leaving out the primary antibody. This slide was mounted with gum for examination and capture by the Olympus BX51 microscopic/DP70 Digital Camera System for study comparison.

**Table 1:** NRIP expression in 48 cores of tumors in US Biomax BCN962 tissue microarray

ID	Organ type	Histological diagnosis	Differentiation	IHC score/NRIP expression
A	Esophagus			
	A1-3	Squamous cell carcinoma	Poorly	6/+, 8/++, 0/-
B	Stomach			
	B1	Adenocarcinoma	Moderately	0/-
	B2	x		0/-
	B3	Mucinous Adenocarcinoma	Poorly	0/-
C	Colon			
	C1-2	Mucinous Adenocarcinoma	Moderately	6/+, 4/+
	C3	x		0/-
D	Prostate			
	D1-2	Adenocarcinoma	Gleason's grade 3+4	0/-, 0/-
	D3	Adenocarcinoma	Gleason's grade 4+5	0/-
E	Liver			
	E1-2	Hepatocellular carcinoma	Grade II	0/-, 0/-
	E3	Hepatocellular carcinoma	Grade III	0/-
F	Lung			
	F1-2	Adenocarcinoma	Moderately	0/-, 0/-
	F3	Squamous cell carcinoma	Moderately	0/-
G	Kidney			
	G1-2	Renal cell carcinoma	Conventional type	0/-, 0/-
	G3	Nephroblastoma		0/-
H	Breast			
	H1-3	Infiltrating ductal carcinoma	Grade II	6/+, 0/-, 0/-
I	Uterine cervix			
	I1-3	Squamous cell carcinoma (Keratinized)	Moderately	0/-, 1/-, 0/-
J	Ovary			
	J1-3	Serous Adenocarcinoma	Grade III	9/++, 12/+++, 0/-
K	Urinary Bladder			
	K1, K3	Transitional cell carcinoma	Grade II	0/-, 0/-
	K2	Transitional cell carcinoma	Grade III	0/-
L	Lymph node			
	L1-3	Large B-cell lymphoma		1/-, 2/-, 0/-
M	Skin			
	M1-2	Squamous cell carcinoma	Well	1/-, 0/-
	M3	Squamous cell carcinoma	Poorly	4/+
N	Pancrease			
	N1, N3	Ductal Adenocarcinoma	Poorly	4/+, 0/-
	N2	Acinar cell carcinoma		9/++
O	Testis			
	O1-2	Seminoma		0/-, 0/-
	O3-4	x		0/-, 0/-
	O5	Embryonal rhabdomyosarcoma		0/-
P	Tongue			
	P1	Alveolar rhabdomyosarcoma		0/-

1. IHC scores/NRIP expression: 10–12/+++ (Strong), 7–9/++ (intermediate), 3–6/+ (weak), 0–2/- (negative or low expression not exceed the cut-off threshold of 3).

2. x: tumor-sparing normal tissues

Although a number of computer-based programs were designed specifically for the quantitative analysis of IHC, there still seemed to lack of generally wide acceptance in research laboratories and clinical practices for evaluation of the immunoreactivity and histochemical appearance. Their resultant objective accuracy did not significantly improved clinical outcome measures, compared with the conventional analysis by pathologists [7-9]. In this study, the tissue microarray (TMA) slides were simultaneously reviewed and scored by 2 qualified pathologists (CP Han & LF Kao) with agreements, by using a two-headed microscope. IHC nuclear scoring algorithm has not been optimized and standardized, so scores were given based on a semi-quantitative scoring system developed for this study. NRIP expression was quantified after evaluating both the intensity of the reaction and the extent of the reaction. The intensity of NRIP expression was quantified using the following scores: 0 = negative, 1 = weakly positive, 2 = moderately positive, 3 = strongly positive. The extent of NRIP expression was quantified by evaluating the percentage of the positive staining areas in relation to the whole cancer areas in the core, where a score of 0 was given for 0% reactivity, 1 point was assigned for 1–10% reactivity, 2 points were assigned for 11–50% reactivity, 3 points were given for 51–80% reactivity, and samples with >80% reactivity were assigned a total of 4 points. The final immunoreactive score was determined by multiplying the intensity score by the extent score, with the minimum score attainable being 0 and a maximum score of 12. The 12-tier scoring was also simplified by combining scores 10–12: strong NRIP expression (+++), 7–9: intermediate NRIP expression (++), 3–6: weak NRIP expression (+), and 0–2: negative NRIP expression (-). ROC curve analysis was used in the selection of the optimal cut-off for determining threshold for NRIP positivity [10,11]. The cut-off threshold was set as 3 for this interpretation by the best sensitivity and specificity (maximum sum of sensitivity and specificity). Score of 3 points or greater was considered positive for NRIP expression.

### Statistical Methods

Restricted to limited numbers of tissues from a variety of organ groups, descriptive statistics will be mainly used following our data description. Fisher's exact test was used to calculate the sensitivity and specificity of NRIP expression based on results obtained from the study sample and control group. The sensitivity of NRIP expression is defined as the proportion of positive NRIP expression among the known tumor tissues. The specificity of NRIP expression is defined as the proportion of negative NRIP expression among the control group of matched and unmatched non-neoplastic tissue samples.

## Results

**Figure 2:**
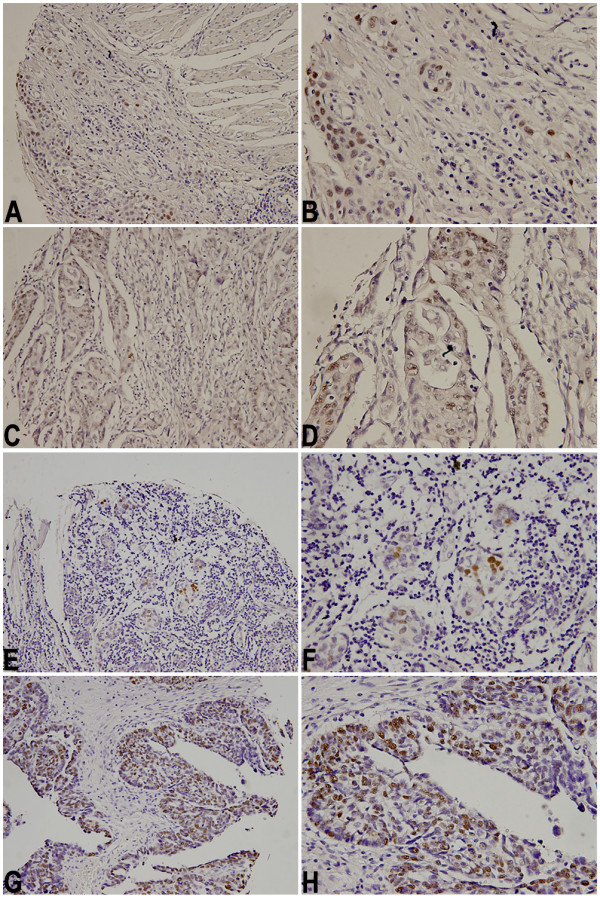
**Immunohistochemistry demonstrated NRIP expression at subcellualr nuclear localizations**. A, B: esophageal squamous cell carcinoma in core A2 specimen, C, D: colon adenocarcinoma in core C2 specimen, E, F: breast adenocarcinoma in core H1 specimen, and G, H: ovarian adenocarcinoma in core J2 specimen. A, C, E, G photomicrographs were taken in middle-powered, ×200; B, D, F, H photomicrographs were taken in high-powered, ×400.

The study shows that NRIP expression can be detected in certain types of tumors (Figure 2) Forty-eight tumor cores were examined from 16 different organs: esophagus, stomach, colon, prostate, liver, lung, kidney, breast, uterus, ovary, bladder, lymph node, skin, pancreas, testis, and tongue. Of the 48 tumor cores, 44 cores revealed tumor tissue consistent with the histopathologic diagnostic neoplastic labeling after examination by two board-certified pathologists. The remaining 4 cores (one gastric: B2, one colonic: C3, and two testicular: O3-4) did not reveal any cancerous cells on our pathological review. These 4 tumor-sparing cores of tissues contained normal organ areas or fibro-adipose components. The IHC staining results of these 48 cores showed that some types of specific cancer cells were positive, but others were negative for monoclonal antibodies raised against human NRIP. The ID (A-P), organ type, histological diagnosis, differentiation, IHC scores (0–12) and NRIP expression status (-, +, ++, +++) are shown in Table 1 for each groups of cancers.

The study also demonstrates that NRIP is not strongly expressed in non-neoplastic tissue. NRIP expression was evaluated in a control group of 48 cores obtained from matched or unmatched tissue from 16 different organs: esophagus (a1-3), stomach (b1-3), colon (c1-3), prostate (d1-3), liver (e1-3), lung (f1-3), kidney (g1-3), breast (h1-3), Uterine cervix (i1-3), ovary (j1-3), bladder (k1-3), stomach lymph node (l1-3), skin (m1-3), pancreas (n1-3), testis (o1-4), and placenta (q1-2). In this preliminary study, NRIP seemed to be stained weakly in a limited extent of b1 (normal gastric mucosal epithelium with IHC score of 1), d1 (normal prostate glandular epithelium with IHC score 2), g2 (normal renal tubular epithelium with IHC score of 1), m1 (normal skin squamous epithelium with IHC score of 1, which was restricted to basal cell layers), and n3 (normal pancreatic acinar epithelium with IHC score 2). All these IHC scores were beneath the cut-off threshold of 3, so the NRIP expression results were considered as negative in each of these groups. Each kind of the major indigenous cell types in these healthy non-pathologic tissues, such as other specific epithelial cells, endothelial cells, germ cells, blood cells and nerve cells etc were also stained minimally with IHC score below the cut-off.

### Statistical analysis

NRIP expression was not detected above an IHC score of 3 in any of the matched or unmatched healthy non-neoplastic tissue of the control group, resulting in 100% NRIP expression specificity. However, of the 44 cores of neoplastic tissue confirmed by histopathological re-evaluation, only 10 cores of tumor revealed positive NRIP expression (IHC cut-off score ≧ 3). Overall NRIP expression sensitivity in this group was only 10/44 = 23%.

For subsequent analysis, we also regrouped all of the tissue samples into two categories: hormone-related tumor and primary carcinoma. Among the 9 hormone-related tumors, 3 cores of tumor revealed positive NRIP expression and therefore 33% sensitivity. In the 36 primary carcinoma tumor samples, 10 cores of tumor revealed positive NRIP expression with a comparable sensitivity of 28% for NRIP expression in this subgroup. Fisher's Exact test showed a p-value of 0.010 (<0.05) between primary carcinoma tumor and matched controlled tissues. This indicated that there was a significant difference in positive NRIP expression between primary carcinoma tissue and matched controlled healthy tissue cores.

## Discussion

Transcriptional regulation by members of the nuclear hormone receptor super-family is a complicated process requiring the mediation of distinct subclasses of co-regulators. Nuclear receptors function as ligand-inducible transcription factors. The binding of growth hormonal ligand to nuclear receptors can induce receptor dimerization, facilitating the ability of the nuclear receptor to bind to its cognate responsive element and recruit co-regulators to promote the expression of target genes. NRIP interacts with nuclear receptors (such as AR and GR), and trans-activates the distinct promoters in vitro [1,2]. We have already known that steroid hormones, acting as promoters, were involved in carcinogenic process in some tumor types, such as breast, ovary, testis, prostate, uterine endometrium and thyroid [12]. NRIP, acting as one nuclear transcriptional regulator of the steroid receptors, may be a critical target for developing diagnostic or therapeutic agents against nuclear receptors mediated progression of some types of cancers.

The tissue microarray (TMA), which facilitates rapid translation of molecular discoveries to clinical applications, has become a useful tool in translational research. It also permits us to complete studies that previously spanned months and comprised hundreds of whole tissue sections now in a matter of days on one microscope glass slide [12,13]. In this study, by using the commercially derived human tissue microarray (TMA) (US Biomax Inc, Catolog No. BCN 962), which contained 96 cores including 48 types of primary tumors, as well as 48 matched or unmatched non-neoplastic controlled tissues, we attempted to investigate the in situ localization and expression status of this newly discovered NRIP gene in various human tissues.

Immunohistochemistry (IHC) has become an important tool in the biomedical and histopathological research. Although some scoring systems, such as the H-score, Allred Score etc, have attempted to incorporate both tumor cell staining percentages and nuclear staining intensity of estrogen receptor (ER) and progesterone receptor (PR) into a single total score in breast cancer, the IHC nuclear scoring analysis algorithm has not yet been fully optimized for dissimilar biomarkers on various tissue types. Different laboratories may use different scoring schemes for the nuclear stains. For example, some laboratories use only the percentage of positive nuclei as a score and use different cut-off thresholds of 1%, 5% or 10% for the interpretation [6-8,14-18]. Instead of the binary positive-negative end point, we used a 4-point scale (0, 1, 2, 3) to distinguish the staining intensity. The final IHC score for this newly discovered NRIP expression in human tissues was calculated by multiplying the average staining intensity times the percentage of the positive staining areas in relation to the whole cancer areas. A combination of 3 or greater was considered positive for NRIP expression.

The development of tissue microarray (TMA) technology provides methodology for high-throughput concomitant analyses of the patterns of newly cloned gene expression on large numbers of archival tumor and non-tumor tissue samples. After carefully examining the 96 cores of samples by two board-certified pathologists (LF Kok and CP Han), we ascertained that the subcellular compartmentalization of NRIP gene expression was only restricted to the nucleus, not in the cytoplasm or cell membrane. This IHC results provided further evidence to support and demonstrate Prof. Chen's earlier suggestion that NRIP may be a nuclear localizing protein.

In this study, we found that some cases of squamous cell carcinomas or adenocarcinomas can be stained positively for NRIP, however, others cannot. Not all cases of specific tumors consistently revealed positive NRIP expression. Of the 48 cores of tumors from US Biomax BCN 962 TMA, ten cores of tumors (two cases of esophageal squamous cell carcinoma, two cases of colon adenocarcinoma, one case of breast adenocarcinoma and two cases of ovarian adenocarcinoma, one case of skin squamous cell carcinoma, one cases of pancreatic ductal adenocarcinoma, and one case of pancreatic acinar cell carcinoma) revealed positive NRIP expression. The other 38 tumor cores including 4 tumor-sparing non-pathological tissues did not reveal NRIP expression. On the other hand, NRIP was not expressed in any of the 48 cores of matched or unmatched controlled non-neoplastic healthy tissues. Among the 9 steroid hormone related tumors, we found that one breast and two ovarian cancer tissues showed positive NRIP nuclear staining, while prostate and testicular cancer did not. However, after using another tissue microarray of prostate cancers only, the results showed positive NRIP nuclear staining in some cases. (Data not shown)

Based on the results of the study, the sensitivity and specificity of NRIP expression was calculated in hopes of delineating the utility of IHC scoring of NRIP expression as a potential adjunct for cancer diagnosis. The sensitivity of positive NRIP expression in neoplastic tissues was only 23%, indicating a high false-negative rate. The negative predictive value was 58.6%, also meaning that if the IHC NRIP expression is negative, there is still a high probability that the cancer tissue diagnosis will be missed. It is possible that the tissue consists of tumor cells that do not express NRIP. Hence, NRIP expression may not be a good IHC tool for cancer screening. The specificity of negative NRIP expression in non-neoplastic healthy tissues was 100%, indicating a low false-positive rate. The positive predictive value was also 100%, meaning that if the IHC NRIP expression is positive, there is a high probability that the tissue in question is truly neoplastic. Hence, NRIP expression may be a good IHC tool for cancer confirmation. NRIP's high specificity for cancer cells and high positive predictive value makes it a useful marker in confirming the presence of carcinogenic tissue in biopsies and tissue extractions. Used in conjunction with a thorough history and physical exam, NRIP can be utilized to as an adjunct to help expediting the diagnosis of cancer.

Our data collectively confirmed that there is a significant difference in positive NRIP expression between carcinoma and matched controlled healthy tissues. It is suggested that NRIP expression to exert its transcriptional activity might play a role in the carcinogenic process of some kinds of tumors by exerting transcriptional activity. The nuclear regulatory protein in neoplastic transforming process might be changed according to the differentiation and maturation of the tumor cells. However, before this relationship can be conclusively delineated, further investigation of NRIP expression in a larger numbers of cases and in various types of tumor cells will need to be performed and clinical correlation will need to be determined.

## Conclusion

This is the first study to use a tissue microarray (TMA) and immunohistochemical (IHC) techniques to investigate the distribution of newly discovered NRIP expression in different human neoplastic and normal controlled tissue types. When using NRIP as a potential diagnostic tool for human malignancies, the low sensitivity (23%) indicates there is a high probability that the cancer tissue diagnosis will be missed. A test with low sensitivity is not useful to exclude a diagnosis because a less sensitive test will render more false-negative results. On the other hand, the high specificity (100%) of NRIP expression can be used to ensure that healthy non-neoplastic tissue is not missed. If the tissue sample has positive NRIP expression, there is low probability that the tissue is non-neoplastic. In this study, we demonstrated that some cases of certain types of carcinoma groups, such as esophageal squamous carcinoma, colon adenocarcinoma, breast adenocarcinoma, ovarian adenocarcinoma, skin squamous cell carcinoma, pancreatic ductal adenocarcinoma, and pancreatic acinar cell carcinoma express NRIP, while sarcoma, lymphoma, and germ cell tumor groups did not. All controlled matched or unmatched non-neoplastic healthy tissues did not express NRIP. NRIP expression increased significantly in carcinoma tissues in comparison with healthy controls. We suggested that NRIP might have a housekeeping function. Activation of the nuclear NRIP trans-regulatory effects might play a role in the human carcinogenic processes. Further large-scale studies designed to determine whether or not there is an association between nuclear expressions of NRIP in other human neoplastic tissue is needed to better delineate its role and association with neoplastic transformations. By assessing its prognostic and predictive value, NRIP may be a potential new candidate biomarker to identify further therapy targets in the battle against cancer.

## List of abbreviations used

NRIP: nuclear receptor interaction protein; TMA: tissue microarray; IHC: immunohistochemistry.

## Authors' contributions

CPH, LFK performed experiments and wrote the manuscript. YWC carried out the immunohistochemical stains. SLC provided monoclonal anti-human NRIP antibody. MYL performed the statistical analysis. PHW, CCY, YST, SLT participated in its design and coordination. TSW edited the draft manuscript. All authors read and approved the final manuscript.
